# Understanding out-of-pocket expenditure in India: a systematic review

**DOI:** 10.3389/fpubh.2025.1594542

**Published:** 2025-06-09

**Authors:** Sagarika Kamath, Jeffin Maliyekkal, S. Elstin Anbu Raj, R. J. Varshini, Helmut Brand, Andria Sirur, Vishwajeet Singh, Vidya Prabhu, Kumar Sumit, Rajesh Kamath

**Affiliations:** ^1^Department of International Health, Care and Public Health Research Institute—CAPHRI, Faculty of Health, Medicine and Life Sciences, Maastricht University, Maastricht, Netherlands; ^2^Department of Social and Health Innovation, Prasanna School of Public Health, Manipal Academy of Higher Education, Manipal, India; ^3^Centre for Evidence-Informed Decision Making, Prasanna School of Public Health, Manipal Academy of Higher Education, Manipal, India; ^4^Department of Healthcare and Hospital Management, Prasanna School of Public Health, Manipal Academy of Higher Education, Manipal, India; ^5^Manipal School of Commerce and Economics, Manipal Academy of Higher Education, Manipal, India; ^6^Directorate of Online Education, Manipal Academy of Higher Education, Manipal, India; ^7^Department of Global Public Health Policy and Governance, Prasanna School of Public Health, Manipal Academy of Higher Education, Manipal, India

**Keywords:** out of pocket expenditure, Ayushman Bharat, publicly funded health insurance, National Sample Survey Office (NSSO), National Family Health Survey (NFHS)

## Abstract

**Introduction:**

Out-of-pocket expenditure (OOPE) constitutes a substantial portion of healthcare costs in India, accounting for 47.1% of the Total Health Expenditure in 2019–20. Despite a decline from previous years, OOPE remains a significant financial burden, contributing to catastrophic health expenditures and impoverishment for households.

**Methods:**

A systematic review was conducted to analyze factors influencing out-of-pocket expenditures (OOPE) in India. The review adhered to predefined inclusion and exclusion criteria. Search terms were tailored to the syntax of each database to maximize retrieval, using combinations of keywords such as “out of pocket,” “India,” and “national survey.” A total of 702 citations were retrieved (PubMed: 185, Web of Science: 183, Scopus: 334), with 316 identified as duplicates. After title and abstract screening of 386 citations, 128 articles were subjected to full-text review, leading to the inclusion of 36 studies. A narrative synthesis and thematic analysis identified determinants of OOPE in the Indian healthcare context, with findings organized in tables and descriptive formats to address study heterogeneity and enhance interpretation.

**Results:**

This systematic and rigorous methodology ensures a comprehensive and reliable understanding of the determinants of OOPE in the Indian healthcare context. Eleven themes emerged from the review: (1) source of care and disease/ condition, (2) place of residence, (3) economic status, (4) components of OOPE, (5) age, (6) gender, (7) strategies for coping with OOPE, (8) educational attainment, (9) OOPE and institutional deliveries, (11) health insurance.

**Discussion:**

India’s heavy reliance on OOPE emphasizes healthcare gaps, necessitating reforms in public investment, insurance, primary care, and affordable access to ensure equity and financial protection. The lack of equitable healthcare financing instigates the challenges, leading to widespread reliance on distress financing methods.

## 1 Introduction

Out of pocket expenditure (OOPE) on healthcare represents a significant challenge in India, affecting the financial stability and health outcomes of households, particularly among vulnerable populations. Achieving Universal Health Coverage (UHC) is a commonly recognized target of health systems worldwide to ensure that populations can access quality health services without financial hardship. Realizing equitable access to healthcare has necessitated a significant increase in global health expenditures. From 2000 to 2019, these expenditures have more than doubled, rising from US $4.2 trillion [constituting 8.3% of global gross domestic product (GDP)] to US $8.5 trillion (9.8% of global GDP). The distribution of global health spending along the income strata is still disparate, with high-income countries contributing roughly 80% of the aggregate, financed primarily by government spending (70%). In comparison, low-income countries have a strong dependence on external aid (29%) and OOPE (44%).

**Figure 1:**
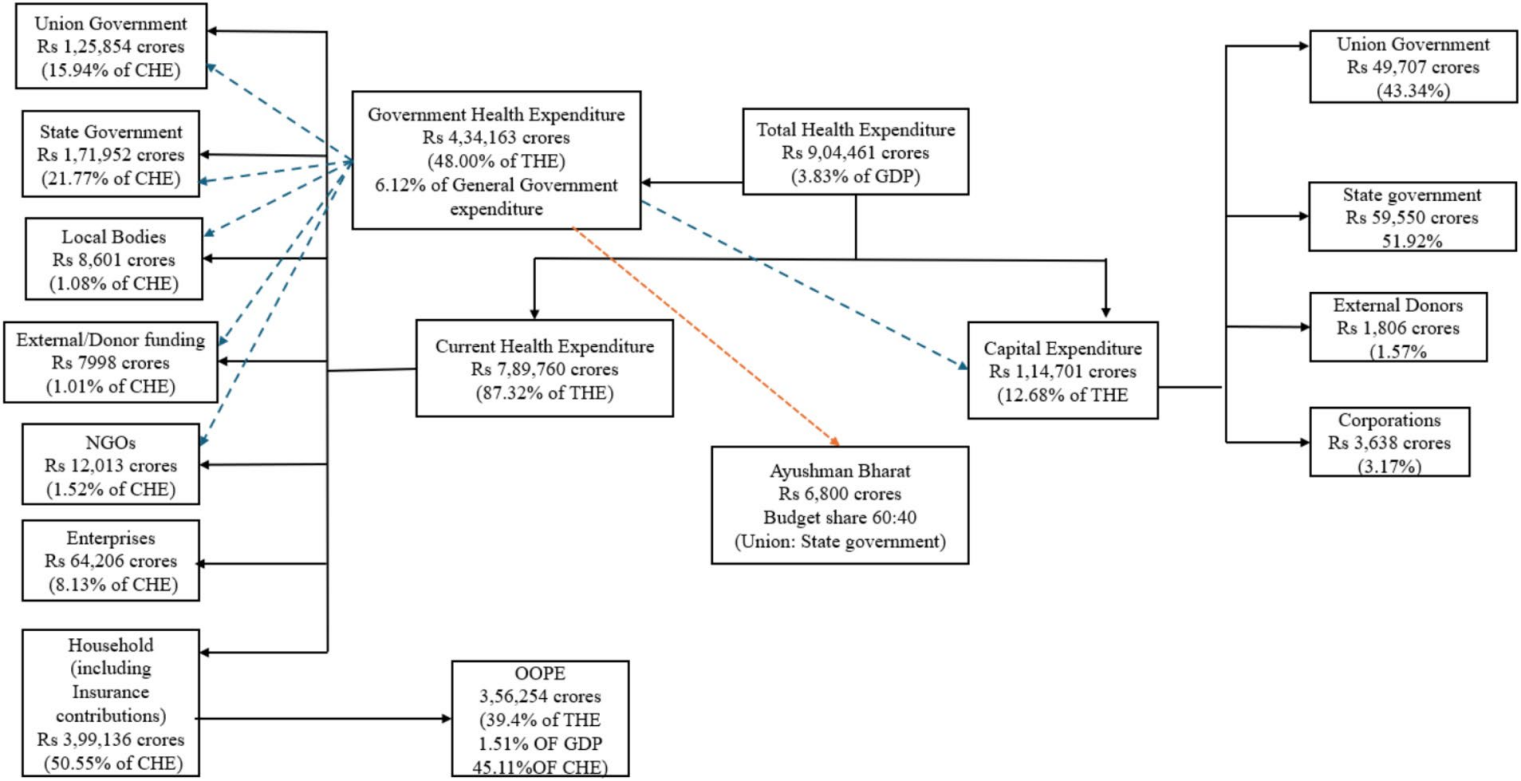
The breakdown for 2021–22.

India’s total health expenditure for 2021–22 is estimated to be Rs. 9,04,461 crores (3.83% of GDP), with OOPE at 47.1% of total health expenditure from 69.4% in 2004–05 (Figure 1). Such a substantial decline in OOPE signifies improved accessibility and affordability of healthcare services by healthcare consumers. OOPE, or the direct payment incurred by the patient upon receiving any healthcare goods or services, is typical in countries with poor governmental commitments for healthcare service provision and the facilitation of risk pooling mechanisms. Moreover, public health spending is not solely dependent on the fiscal capacities of health systems. Prioritizing healthcare spending can be a policy-level issue (1, 2).

In the Indian context, increasing Government Health Expenditure (GHE) to 3% of GDP would reduce OOPE to 30% of overall health expenditure (3, 4). Despite this, India’s public spending on healthcare has been relatively stagnant, from 0.84% of GDP in 2004–05 to 1.84% in 2021–22 (2). This expenditure level is significantly lower than the global average, with other low and low- and middle-income countries (LMICs) allocating approximately 6% of their GDP to public healthcare (5). On the contrary, government spending as a percentage of General Government Expenditure (GGE) depicts a rise from 3.94% in 2014–15 to 6.12% in 2021–22. GHE as a percentage of total health expenditure has also grown from 29% in 2014–15 to 48% in 2021–22. Counterintuitively, the total health expenditure as a share of GDP has decreased from 3.89% in 2014–15 to 3.83% in 2021–22. The total health expenditure per capita (Rs.) at current prices has increased from 3,826 in 2014–15 to 6,602 in 2021–22. The detailed expenditure has been reported in Table 1.

**Table 1:** Comparison of GHE in 2014–15 and 2021–22.

Health expenditure indicator	2014–2015	2021–2022
THE	Rs. 4,83,259 crores	Rs. 9,04,461 crores
THE as a % of GDP	3.89	3.83
Total government health expenditure	Rs. 1,39,949 crores	Rs. 4,34,163 crores
Total government health expenditure (as a % of general government expenditure)	3.94	6.12
Total government health expenditure (as a % of GDP)	1.13	1.60
Total government health expenditure (as a % of THE)	29	48

India’s healthcare system is burdened by several critical shortcomings. These include uneven distribution of healthcare personnel, a weak foundation in primary healthcare, a vast and unregulated private sector, insufficient public health funding, fragmented health data systems, unsustainable rise in medication and technology costs due to irrational use, and inadequate governance and accountability mechanisms (6). While infrastructure has expanded considerably between 2005 and 2020, with increases in subcentres, primary health centers, and community health centers, the distribution of resources remains inequitable across states (7). However, the quality of care offered at public health facilities is often poor and uneven, with many facilities falling short of minimum standards, particularly in less developed states. The public sector’s inability to provide adequate primary care has resulted in a steady decrease in the use of public hospitalization services, especially among wealthier populations, leaving the poor heavily reliant on often substandard public health facilities (8).

Economic policies emphasizing growth since the early 1990s while fostering economic advancement have exacerbated socio-economic disparities in India, contributing to heightened health insecurity (9). The Indian healthcare landscape is characterized by a substantial, heterogeneous, and largely unregulated private sector, which has emerged as a consequence of the public sector’s limited reach. By 2014, private providers dominated both outpatient and inpatient care, particularly in urban areas. This trend is underscored by the private sector’s significant contribution to the increase in hospital beds between 2002 and 2010. Private practitioners have become the primary point of contact for various health concerns across rural and urban settings (10).

The Fairness of Financial Contribution (FFC) index is a measure used to evaluate equity in healthcare financing, with values ranging from zero to one. A perfect equity score of one indicates that all individuals pay the same proportion of their capacity to pay (CTP), while values below one signifies inequality in healthcare payments relative to CTP. The FFC index captures both horizontal and vertical inequities but has limitations in distinguishing between them when households with different CTPs contribute varying proportions of their income to healthcare (11).

In the Indian context, FFC index values for out-of-pocket payments have shown a declining trend from 1993–1994 (0.8851) to 2011–2012 (0.8512), indicating a deterioration in the fairness of healthcare financing and reduced protection for vulnerable populations against excessive out-of-pocket spending. This decline may be attributed to several factors, including the introduction of user charges in public facilities, rising costs of medicines and diagnostic tests, and increased hospitalization charges (Table 2). The healthcare payment structure in India appears to be moving toward lesser fairness in out-of-pocket payments, with low-income groups experiencing a sudden increase in healthcare expenditure between 2009 and 2012. However, it is important to note that while the FFC index provides insight into overall fairness trends, it cannot explicitly explain whether the observed changes are due to horizontal or vertical redistributive effects of health financing. This limitation underscores the need for complementary analyses to fully understand the dynamics of healthcare financing equity in India (12).

**Table 2:** Comparison of OOPE breakdown for 2014–2015 and 2021–22.

Healthcare provider		OOPE for 2014–2015		OOPE for 2021–2022	
		(In ₹ crores)	(% of total OOPE)	(In ₹ crores)	(% of total OOPE)
Hospitals	General hospitals – government	22,429	7.4	14,319	4.01
	General hospitals - private	86,189	28.5	1,20,608	33.85
Providers of ambulatory healthcare	Offices of general medical practitioners	15,760	5.2	17,685	4.96
	Other healthcare practitioners	412	0.14	302	0.08
	All other ambulatory centers	1,645	0.54	1,016	0.28
Providers of ancillary services	Providers of patient transportation and emergency rescue	18,934	6.3	20,103	5.64
	Medical and diagnostic laboratories	20,610	6.8	25,047	7.03
Retailers and other providers of medical goods	Pharmacies	1,30,451	43.1	1,52,842	42.90
	Retail sellers and other suppliers of durable medical goods and medical appliances	559	0.18	837	0.23
Providers of preventive care	Providers of preventive care	4,225	1.4	1,480	0.41
Other healthcare providers not elsewhere classified		1,210	0.4	2,017	0.56
Total		3,02,425		3,56,254	

Heavy reliance on OOPE as its share in CHE or THE places a significant financial burden on a country’s population, leading to higher Catastrophic Health Expenditures and poverty rates. Healthcare spending for approximately 90 million Indians has surpassed the “catastrophic” threshold. This condition is characterized by health expenditures exceeding 10% of household consumption, thereby jeopardizing the household’s ability to meet subsistence needs (13).

In 2021–22, Private Hospitals accounted for Rs. 2,12,948 crores (26.96% of CHE), and Government Hospitals accounted for Rs. 1,49,900 crores (18.99% of CHE) (2). An asymmetrical dependency on health financing strategies at the expense of prioritizing the delivery of high-quality and accessible healthcare undermines the bulk of efforts to manage OOPE. Investing in public healthcare infrastructure, promoting preventive measures, ensuring that financial mechanisms prioritize health outcomes rather than solely focusing on revenue generation, and adopting a patient-centered approach would prove more effective in containing high OOPE. This systematic literature review aims to inquire into the financial, administrative, and clinical dimensions of OOPE, unraveling the intricacies of India’s health financing landscape. By delving into the historical trends and the current state of OOPE in the country, the study aims to provide a nuanced understanding of the elements influencing the current scenario. The results of this review hope to foster a deeper understanding of the challenges and opportunities within India’s health financing landscape to pave the way for informed decision-making and evidence-based policy interventions that prioritize accessibility, quality, and financial sustainability in healthcare.

## 2 Methodology

### 2.1 Inclusion/exclusion criteria

#### 2.1.1 Criteria for including studies in the review

1.  The study should be conducted in India.

This geographically focused approach allows for a targeted analysis of OOPE-specific to the Indian healthcare system and its unique socio-economic context.

2.  The study should be a secondary analysis of any rounds of either National Family Health Survey (NFHS) or National Sample Survey Office (NSSO).

These nationally representative surveys provide robust and comprehensive data on various health and demographic indicators in India, ensuring the generalizability of the findings. Furthermore, these two surveys are also among the data sources leveraged by the National Health Accounts (NHA) for officially capturing healthcare expenditures within the country. This alignment with the NHA’s established methodology strengthens the credibility and generalizability of the findings derived from studies employing these datasets.

3.  At least one of the study’s explicitly stated outcomes must be directly related to OOPE incurred by individuals or households in the Indian healthcare system. This focus on OOPE ensures the direct relevance of the study to the review’s central theme.

#### 2.1.2 Criteria for excluding studies in the review

1. Any work other than original research articles like series, comments, letters, editorials, books, book chapters etc. were excluded.
2. Studies employing NSSO/NFHS data at the state or district level were excluded due to the focus on national-level analysis to ensure data comparability and facilitate the generation of findings applicable to the entire country.

### 2.2 Search methods for identification of studies

To comprehensively identify relevant studies, a systematic search strategy was employed across multiple electronic databases. The following databases were searched from their inception dates until February 23rd, 2024: PubMed, Scopus, and Web of Science. A predefined set of keywords was established before initiating the search process. The initial search was conducted in PubMed and subsequently replicated in the other two databases (Scopus and Web of Science) to ensure consistency.

### 2.3 Search strategy

Due to potential variations in search syntax across platforms, each database utilized a slightly modified search strategy. Details regarding the specific search strategies for each database is given below:

#### 2.3.1 PubMed

((“out of pocket”[Title/Abstract]) AND (India)) AND (National Survey)

Search: ((out of pocket [Title/Abstract]) AND (India)) AND (National Survey).

“out of pocket”[Title/Abstract] AND (“india”[MeSH Terms] OR “india”[All Fields] OR “india s”[All Fields] OR “indias”[All Fields]) AND ((“ethnicity”[MeSH Terms] OR “ethnicity”[All Fields] OR “nationalities”[All Fields] OR “nationality”[All Fields] OR “federal government”[MeSH Terms] OR (“federal”[All Fields] AND “government”[All Fields]) OR “federal government”[All Fields] OR “national”[All Fields] OR “nation”[All Fields] OR “nation’s”[All Fields] OR “nationalism”[All Fields] OR “nationalisms”[All Fields] OR “nationalization”[All Fields] OR “nationalized”[All Fields] OR “nationally”[All Fields] OR “nationals”[All Fields] OR “nations”[All Fields] OR “nations’ s”[All Fields]) AND (“survey s”[All Fields] OR “surveyed”[All Fields] OR “surveying”[All Fields] OR “surveys and questionnaires”[MeSH Terms] OR (“surveys”[All Fields] AND “questionnaires”[All Fields]) OR “surveys and questionnaires”[All Fields] OR “survey”[All Fields] OR “surveys”[All Fields])).

#### 2.3.2 Scopus

(ALL (out AND of AND pocket) AND TITLE-ABS-KEY (india AND national AND survey))

#### 2.3.3 Web of science

(ALL = (Out of Pocket)) AND TS = (India AND National Survey)

### 2.4 Data collection

Result of search strategy was imported to Rayyan systematic review software. Duplicates were detected with the help of the software and manually removed.

### 2.5 Selection of studies

Following deduplication, unique citations were subjected to title and abstract screening. Eligible abstracts of all the relevant studies as per the inclusion criteria were included for full-text screening. The unique citations were exported to Microsoft Excel spreadsheet and relevant ones from these were included for analysis. Subsequently, only open-access or articles with full-text accessibility through institutional subscriptions were included for further analysis. Studies lacking such accessibility were excluded.

### 2.6 Data analysis

Given the heterogeneity of the data, a narrative synthesis approach was employed to address the research question when applicable. For studies with less comparable data, results were thematically synthesized and presented in tables.

### 2.7 Public and patient involvement

We did not involve public or patient during the process of this review.

## 3 Results

The literature search on electronic databases such as PubMed, Scopus, Web of Science generated 702 articles, out of which 316 were duplicates. After title and abstract screening of 386 citations, 128 were included for full-text screening, of which 36 articles were included for data synthesis. A total of 702 records were identified through database searches, including PubMed (*n* = 185), Scopus (*n* = 334), and Web of Science (*n* = 183). After removing 316 duplicate records, 386 records remained for screening. Of these, 258 records were screened for eligibility based on title and abstract. Subsequently, 386 reports were sought for full-text retrieval, and 79 were excluded after full-text review. Reasons for exclusion included fragmented studies (*n* = 28), no access to full text (*n* = 17), outcomes not relevant (*n* = 12), wrong publication type (*n* = 9), wrong survey (*n* = 8), and information being a repetition from other included literature (*n* = 5). Ultimately, 49 full-text articles were included in the final review based on the eligibility criteria.

### 3.1 Characteristics of included studies

The characteristics of the NSSO rounds employed in the selected studies are summarized in Table 3. Two of the 20 studies incorporated data from multiple NSSO rounds.

**Table 3:** NSSO rounds employed by the studies under review.

Survey	Subject	Year	Sample size	Study IDs
NSSO 50th round	Differences in level of consumption among socio-economic groups	1993–1994	1,15,354 households–5,64,537 individuals	(41)
NSSO 52nd round	Morbidity and treatment of ailments	1995–1996	1,20,000 households−6,00,000 individuals	(14, 32)
NSSO 60th round	Morbidity, healthcare and the condition of the aged	2004	73,868 households–3,83,338 individuals	(32, 14, 15, 16, 59, 17, 60, 18, 52, 47, 48)
NSSO 61st round	Household consumer expenditure among socio-economic groups	2004–2005	1,24,680 households–6,02,833 individuals	(41)
NSSO 68th round	Household consumer expenditure	2011–2012	1,00,957 households–4,59,784 individuals	(41)
NSSO 71st round	Social consumption: health	2014	65,932 households−3,33,104 individuals	(41, 14, 15, 52, 60, 57, 58, 19, 34, 20, 61, 43, 21, 62, 33, 53, 49, 38, 35, 39, 45, 54)
NSSO 75th round	Social consumption: health	2017–2018	1,13,823 households–5,55,352 individuals	(15, 52, 43, 22, 40, 23, 24, 25, 26, 27, 28, 29, 30, 37, 63, 55, 56)
NFHS - 4	National family health survey	2015–2016	6,01,509 households–8,03,211 individuals	(42, 31, 44, 46)
NFHS – 5	National family health survey	2019–2021	6,36,699 households–8,25,954 individuals	(42, 50, 51, 36)

This review identified 11 broad factors that influence OOPE in India:

### 3.2 Source of care and disease/condition

Eighteen of the 36 studies selected for review explicitly addressed OOPE for various disease conditions, as detailed in Table 4. OOPE for various disease conditions incurred by households and individuals was reported to be higher in association with private facilities than public facilities. Households in India seeking outpatient care from Informal Health Providers (IHP) primarily address infectious diseases (ID) (67%) compared to non-communicable diseases (NCD). The reliance on IHPs for receiving treatment is significantly higher in rural areas compared to urban areas (22%). Cough, cold, and fever constituted over 80% of ID consultations. Conversely, hypertension, diabetes, and musculoskeletal conditions formed roughly 60% of NCD consultations. Nearly all households incurred OOPE for these IHP services. Non-medical expenditures like travel were negligible, suggesting the localized nature of these consultations. However, direct medical expenses like consultation fees and medications comprised approximately 80% of OOPE, with diagnostic services incurring minimal expenditure (14–31).

**Table 4:** OOPE by disease/condition and service provider.

Disease/Condition		Study ID	NSSO round	Average OOPE per hospitalization (In ₹)				Average OOPE per out-patient visit (In₹)			
				Private	Public	NGO	No care/informal care	Private	Public	NGO	No care/informal care
Child delivery care		(31)	NFHS - 4	10,000*	20*	_	_	_	_	_	_
Hypertension		(28)	75th round	24,565	3,491	21,327	_	576	277	482	66
NCD		(30)	75th round	51,243	13,170	_	_	_	_	_	_
Non-NCDs				32,641	6,245	_	_	_	_	_	_
CVD		(17)	61st round	12,317				_	_	_	_
Diabetes				5,925				_	_	_	_
NCD		(15)	60th round	34,952	14,178	_	_	_	_	_	_
Cancers				49,564				_	_	_	_
CVD				36,347				_	_	_	_
Stroke				33,255				_	_	_	_
Diabetes				20,337				_	_	_	_
Chronic respiratory diseases				12,006				_	_	_	_
Musculoskeletal Disorders				25,900				_	_	_	_
Neuropsychiatric disorders				19,814				_	_	_	_
Genitourinary diseases excluding renal failure				24,018				_	_	_	_
Vision loss and other sensory organ impairments				11,250				_	_	_	_
Others				24,071				_	_	_	_
All NCDs				26,677				_	_	_	_
NCD			71st round	43,052	13,061	_	_	_			
Cancers				78,455				_	_	_	_
CVD				44,406				_	_	_	_
Stroke				55,573				_	_	_	_
Diabetes				21,289				_	_	_	_
Chronic respiratory diseases				18,896				_	_	_	_
Musculoskeletal disorders				31,205				_	_	_	_
Epilepsy				19,698				_	_	_	_
Neuropsychiatric disorders				26,809				_	_	_	_
Genitourinary diseases excluding renal failure				34,719				_	_	_	_
Vision loss and other sensory organ impairments				14,732				_	_	_	_
Others				23,965				_	_	_	_
All NCDs				32,330				_	_	_	_
NCD			75th round	45,393	9,092	_	_	_			
Cancers				70,504				_	_	_	_
CVD				38,837				_	_	_	_
Stroke				41,276				_	_	_	_
Diabetes				20,807				_	_	_	_
Chronic respiratory diseases				17,634				_	_	_	_
Musculoskeletal disorders				34,421				_	_	_	_
Epilepsy				16,819				_	_	_	_
Neuropsychiatric disorders				26,475				_	_	_	_
Genitourinary diseases excluding renal failure				31,924				_	_	_	_
Vision loss and other sensory organ impairments				15,895				_	_	_	_
Others				20,896				_	_	_	_
All NCDs				30,577				_	_	_	_
CD		(16)	60th round	_	_	_	_	280.5	197.7	_	_
NCD				_	_	_	_	345.4	215.6	_	_
Others				_	_	_	_	314.7	223.1	_	_
Rheumatic diseases		(20)	71st round	17,014				622			
Mental health disorders		(29)	75th round	37,152	7,947	_	_	2,358	544	_	_
Cancer		(27)	75th round	1,20,726				4,349			
Multimorbid cancer				74,200				2,374			
Diabetes				26,622				802			
Multimorbid diabetes				48,393				655			
Hypertension				20,397				538			
Multimorbid hypertension				43,876				558			
CVD				69,587				1,417			
Multimorbid CVD				59,821				750			
Neurologic disorders				48,226				1,441			
Multimorbid neurologic disorders				55,170				935			
Genitourinary disorders				40,483				1,841			
Multimorbid genitourinary disorders				60,447				1,126			
NCD				39,900				880			
Multimorbid NCD				48,156				720			
Diabetes		(26)	75th round	2,139.6	459.8	_	_	1,760.3	690	_	_
CVD		(25)	75th round	782	4,791	_	_	1,651	905	_	_
Cancer		(24)	75th round	9,926	2,607	_	_	6,390	11,346	_	_
Cancer		(23)	75th round	71,798	27,504	_	_	99,059	90,429	_	_
Cancer		(19)	71st round	84,320	29,066	_	_	_	_	_	_
Cancer		(22)	75th round	1,15,771	38,859.07	_	_	4,409	2,663	_	_
High – expenditure chronic ailments				55,310	14,078	_	_	884	500	_	_
All chronic ailments				45,169	11,345.22	_	_	871	554	_	_
Diarrhea		(21)	71st round	9,412	2,205	_	_	_	_	_	_
Fever				11,316	3,142	_	_	_	_	_	_
Cataract				13,475	2,191	_	_	_	_	_	_
Tuberculosis				24,154	6,678	_	_	_	_	_	_
Respiratory diseases				16,555	8,163	_	_	_	_	_	_
Asthma				21,218	5,095	_	_	_	_	_	_
Hypertension				20,523	4,122	_	_	_	_	_	_
Diabetes				19,820	5,544	_	_	_	_	_	_
Jaundice				20,928	13,070	_	_	_	_	_	_
Gastro-intestinal diseases				24,311	6,449	_	_	_	_	_	_
Neurological diseases				24,510	9,889	_	_	_	_	_	_
Musculoskeletal diseases				29,021	9,741	_	_	_	_	_	_
Genitourinary diseases				28,622	11,463	_	_	_	_	_	_
Injuries				37,359	8,689	_	_	_	_	_	_
Heart diseases				55,479	15,011	_	_	_	_	_	_
Cancer				76,375	28,281	_	_	_	_	_	_
All diseases				26,407	7,583	_	_	_	_	_	_
Communicable diseases				15,216	4,455	_	_	_	_	_	_
NCDs				36,902	12,301	_	_	_	_	_	_
Inpatient survivors		(14)	52nd round	10,235	4,388	_	_	_	_	_	_
Inpatient decedents				18,357	9,548	_	_	_	_	_	_
Inpatient survivors			60th round	20,208	8,325	_	_	_	_	_	_
Inpatient decedents				31,425	14,043	_	_	_	_	_	_
Inpatient survivors			71st round	26,563	7,361	_	_	_	_	_	_
Inpatient decedents				64,127	18,690	_	_	_	_	_	_
	Certain infectious and parasitic diseases		52nd round	5,950				_	_	_	_
	Neoplasms			21,535				_	_	_	_
	Diseases of blood and blood forming organs			8,572				_	_	_	_
	Endocrine, nutritional and metabolic diseases			8,498				_	_	_	_
Disease-specific expenditure of inpatient survivors classified by ICD 10	Mental and behavioral diseases			5,037				_	_	_	_
	Diseases of eye and adnexa			33,173				_	_	_	_
	Diseases of the circulatory system			11,513				_	_	_	_
	Diseases of the respiratory system			8,637				_	_	_	_
	Diseases of the digestive system			10,539				_	_	_	_
	Diseases of musculoskeletal system & connective tissue			6,617				_	_	_	_
	Diseases of genitourinary system			20,236				_	_	_	_
	Symptoms, signs, and abnormal clinical and laboratory not elsewhere classified			20,357				_	_	_	_
	External causes of morbidity and mortality			9,538				_	_	_	_
	Certain infectious and parasitic diseases		60th round	17,441				_	_	_	_
	Neoplasms			43,431				_	_	_	_
	Diseases of blood and blood forming organs			15,284				_	_	_	_
	Endocrine, nutritional and metabolic diseases			28,456				_	_	_	_
	Mental and behavioral diseases			37,167				_	_	_	_
	Diseases of eye and adnexa			8,311				_	_	_	_
	Diseases of the circulatory system			19,785				_	_	_	_
	Diseases of the respiratory system			8,179				_	_	_	_
	Diseases of the digestive system			46,944				_	_	_	_
	Diseases of musculoskeletal system & connective tissue			17,420				_	_	_	_
	Diseases of genitourinary system			29,953				_	_	_	_
	Symptoms, signs, and abnormal clinical and laboratory not elsewhere classified			24,464				_	_	_	_
	External causes of morbidity and mortality			20,547				_	_	_	_
	Certain infectious and parasitic diseases		71st round	20,068				_	_	_	_
	Neoplasms			66,684				_	_	_	_
	Diseases of blood and blood forming organs			16,086				_	_	_	_
	Endocrine, nutritional and metabolic diseases			22,931				_	_	_	_
	Mental and behavioral diseases			37,167				_	_	_	_
	Diseases of eye and adnexa			16,453				_	_	_	_
	Diseases of the circulatory system			55,267				_	_	_	_
	Diseases of the respiratory system			17,418				_	_	_	_
	Diseases of the digestive system			41,585				_	_	_	_
	Diseases of musculoskeletal system & connective tissue			34,123				_	_	_	_
	Diseases of genitourinary system			46,429				_	_	_	_
	Symptoms, signs, and abnormal clinical and laboratory not elsewhere classified			24,464				_	_	_	_
	External causes of morbidity and mortality			58,696				_	_	_	_
Inpatient survivors	Communicable diseases	(18)	60th round	7,520				_	_	_	_
	Gastro-intestinal diseases			15,577				_	_	_	_
	Febrile Illness			6,826				_	_	_	_
	Tuberculosis			7,603				_	_	_	_
	Other CDs			2,715				_	_	_	_
	Non-communicable diseases			11,564				_	_	_	_
	Cardiovascular diseases			9,137				_	_	_	_
	Diabetes Mellitus			17,006				_	_	_	_
	Bronchial asthma			5,199				_	_	_	_
	Neurological disorders			6,566				_	_	_	_
	Disease of kidney/urinary system			15,649				_	_	_	_
	Accidents/injury/burns/fractures/poison			9,489				_	_	_	_
	Cancer/other tumors			20,058				_	_	_	_
	Other NCDs			4,826				_	_	_	_
	Other diseases and disabilities			8,797				_	_	_	_
Inpatient decedents	Communicable diseases			4,323				_	_	_	_
	Gastro-intestinal diseases			3,636				_	_	_	_
	Febrile Illness			2,915				_	_	_	_
	Tuberculosis			7,060				_	_	_	_
	Other CDs			6,612				_	_	_	_
	Non-communicable diseases			10,604				_	_	_	_
	Cardiovascular diseases			14,201				_	_	_	_
	Diabetes mellitus			6,505				_	_	_	_
	Bronchial asthma			4,102				_	_	_	_
	Neurological disorders			12,153				_	_	_	_
	Disease of kidney/urinary system			11,383				_	_	_	_
	Accidents/injury/burns/fractures/poison			9,609				_	_	_	_
	Cancer/other tumors			18,225				_	_	_	_
	Other NCDs			7,832				_	_	_	_
	Other diseases and disabilities			6,137				_	_	_	_
IP survivors	Inpatient survivors		60th round	9,319	3,829	_	_	_	_	_	_
	Non-communicable diseases			10,604				_	_	_	_
	Communicable diseases			4,323				_	_	_	_
	Other diseases and disabilities			6,138				_	_	_	_
IP decedents	Inpatient decedents			14,151	6,212	_	_	_	_	_	_
	Non-communicable diseases			11,564				_	_	_	_
	Communicable diseases			7,520				_	_	_	_
	Other diseases and disabilities			8,798				_	_	_	_

*Median OOPE.

A longitudinal analysis of household OOPE reveals a growing burden attributable to NCDs. The proportion of spending dedicated to NCDs increased from 31.6% in 1995–1996 to 47.3% in 2004, highlighting the escalating financial strain placed on households by these conditions. Furthermore, within the NCD category, OOPE was particularly high for hospitalizations and outpatient visits associated with cancer, heart disease, and injuries. Medications, diagnostic tests, and medical devices constituted nearly half of all out-of-pocket healthcare spending (32). Additionally, households with NCDs experience a greater burden of OOPE compared to those without (30).

#### 3.2.1 OOPE and end-of-life care for deceased patients

The financial consequences of in-hospital mortality in terms of OOPE was examined, there was a significant rise in inpatient spending for deceased patients, particularly within the middle-aged demographic, and a decline with further advancement in age. This trend is likely attributable to the high costs associated with treating terminal illnesses, as evidenced by the prevalence of diagnoses related to neoplasms (cancers), the circulatory system (heart disease), the genitourinary system, and external causes of morbidity (accidents and injuries) among deceased inpatients. The mean inpatient expenditure for deceased patients increased by 94% between 2004–2005 and 2014–2015, compared to a 26% increase for survivors. This disparity is further amplified by the higher costs associated with private hospitals for deceased patients. In 2014–2015, the mean inpatient expenditure for deceased patients was nearly double that of survivors. Furthermore, controlling for other factors, inpatient spending for deceased patients continued to rise significantly over time, while the gap in out of pocket (OOP) inpatient costs between survivors and deceased patients widened (14, 18).

### 3.3 Place of residence

Eight studies included in this review investigated the association between place of residence and OOPE for NCDs as shown in Table 5. Urban residents reported higher out-of-pocket expenses for non-communicable diseases than rural residents, in terms of place of residence. In the rural sector, the total OOPE incurred at private facilities is approximately 1.5 to 2 times higher compared to public facilities. This disparity is even more pronounced in urban areas, where private facilities exhibit OOPE levels 2–4 times greater than public facilities.

**Table 5:** OOPE by disease/condition and place of residence.

Disease/Condition	Study ID	Survey	Average OOPE per hospitalization (In ₹)				Average OOPE per out-patient visit (In ₹)			
			Rural		Urban		Rural		Urban	
			Private	Public	Private	Public	Private	Public	Private	Public
Mental health disorders	(29)	75th round	37,152	7,947	_	_	2,358	544	_	_
CVD	(25)	75th round	2,690		3,693		1,220		1,573	
Cancer	(24)	75th round	6,559		6,532		9,091		8,392	
Cancer	(23)	75th round	53,597		48,677		1,00,484		82,401	
NCD	(34)	71st round	33,157	10,487	50,614	12,183	703	449	908	401
Cancer	(19)	71st round	77,903	32,202	94,443	24,044	_	_	_	_
Institutional delivery	(36)	NFHS - 5	23,914*	2,039*	28,417*	2,067*	_	_	_	_
Diarrhea	(21)	71st round	4,471		7,295		_	_	_	_
Fever			7,857		9,109		_	_	_	_
Cataract			6,783		16,229		_	_	_	_
Tuberculosis			11,451		17,181		_	_	_	_
Respiratory diseases			12,136		16,387		_	_	_	_
Asthma			13,217		14,721		_	_	_	_
Hypertension			14,132		14,560		_	_	_	_
Diabetes			14,082		16,571		_	_	_	_
Jaundice			13,219		24,725		_	_	_	_
Gastro-intestinal diseases			15,645		23,389		_	_	_	_
Neurological diseases			16,478		22,300		_	_	_	_
Musculoskeletal diseases			18,228		32,387		_	_	_	_
Genitourinary diseases			22,105		27,921		_	_	_	_
Injuries			22,474		30,531		_	_	_	_
Heart diseases			34,589		49,529		_	_	_	_
Cancer			56,305		58,712		_	_	_	_
All diseases			16,558		24,107		_	_	_	_
Communicable diseases			9,236		13,456		_	_	_	_
NCDs			25,182		33,892		_	_	_	_
Inpatient survivors	(18)	60th round	6,144		10,025		_	_	_	_
Inpatient decedents			9,294		10,059		_	_	_	_
Child delivery care	(31)	NFHS - 4	600*		1500*		_	_	_	_
Child delivery care	(35)	71st round	6,851		12,384		_	_	_	_

*Median OOPE.

Rural–urban disparities are evident in the intensity of OOP health expenditure, measured as both a share of total consumption expenditure (TCE) and average per capita expenditure. The findings reveal that the rural population allocates a larger portion of their TCE toward healthcare, while urban areas experience higher average per capita expenditure on healthcare. This pro-rich bias in the intensity of OOPE, observed in both rural and urban settings, can likely be attributed to the principle that the financial burden of OOPE increases alongside an individual’s capacity to pay. Urban areas demonstrate a pattern where the burden of OOPE is concentrated on lower-income groups. In contrast, rural areas exhibit a pro-rich disparity, particularly at higher expenditure thresholds (33). In the context of inpatient care, economically disadvantaged urban residents exhibit a significantly higher concentration of distress financing methods compared to their rural counterparts. Conversely, for outpatient care, the incidence of such distress financing is more prevalent among the rural poor (18, 19, 21, 23–25, 29, 31, 34–36).

### 3.4 Economic status

Fourteen studies included in this review examined the influence of socioeconomic status on OOPE as detailed in Table 6. The influence of socioeconomic status on OOPE was examined, and the studies employed a stratification approach based on the five MPCE (Monthly *Per Capita* Expenditure) quintiles. This approach categorized the study population into five socioeconomic groups, with Q1 representing the lowest income group (poorest) and Q5 representing the highest income group (richest). The interaction between socioeconomic status and healthcare provider type on OOPE, the combined effects of socioeconomic status, type of healthcare provider, and place of residence on OOPE demonstrated a trend of higher relative OOPE (as a proportion of income) for individuals with higher socioeconomic status, revealing a progressive pattern. However, when examining the burden of OOPE as a proportion of income, the data suggests that the poorest quintile dedicates a larger share of their earnings to healthcare compared to the wealthiest quintiles (37). The cost of hospitalization due to childbirth also exhibits a substantial disparity between income quintiles, with the richest spending six times more than the poorest (38).

**Table 6:** OOPE by disease/condition and economic status.

Disease/Condition	Study ID	Survey	Sector		Average OOPE per hospitalization (In ₹)					Average OOPE per out-patient visit (In ₹)				
					Q1 (Poorest)	Q2	Q3	Q4	Q5 (Richest)	Q1 (Poorest)	Q2	Q3	Q4	Q5 (Richest)
Child delivery care	(31)	NFHS - 4	_		500*	500*	500*	1000*	5000*	_	_	_	_	_
Child delivery care	(35)	71st round	_		3,967	6,078	7,304	9,305	15,361	_	_	_	_	_
Child delivery care	(39)	71st round	Private		8,045	15,125	18,841	16,111	42,815	_	_	_	_	_
			Public		3,851	2,587	4,932	3,796	4,880	_	_	_	_	_
Institutional delivery	(36)	NFHS - 5	Private		18,926*	20,328*	23,795*	26,321*	30,300*					
			Public		1,771*	2,067*	2,214*	2,255*	2,114*					
Hypertension	(28)	75th round	_		5000*	4650*	7150*	5200*	7550*	_	_	_	_	_
NCD	(30)	75th round	Private		30,894	43,467	47,909	47,004	67,097	_	_	_	_	_
			Public		9,722	9,204	11,554	13,577	20,541	_	_	_	_	_
Non-NCD			Private		26,585	31,570	31,884	29,636	38,013	_	_	_	_	_
			Public		5,774	6,770	6,101	6,188	6,399	_	_	_	_	_
CVD	(17)	61st round	_		Poorest 40% 5,568		Middle 40% 9,203	Richest 20% 17,431		_	_	_	_	_
Diabetes					Poorest 40% 4,152		Middle 40% 5,106	Richest 20% 6,959		_	_	_	_	_
NCD	(15)	60th round	_		16,076	18,942	19,374	26,666	42,766	_	_	_	_	_
		71st round			19,002	20,441	25,002	31,337	56,966	_	_	_	_	_
		75th round			20,771	25,135	20,608	24,474	39,472	_	_	_	_	_
Mental health disorders	(29)	75th round	Private		59,502	38,767	28,316	26,633	35,036	1,094	815	6,405	1,098	1,483
			Public		12,798	5,837	3,785	6,280	9,591	609	709	757	234	445
CVD	(25)	75th round	_		1,478	2,592	2,773	3,096	5,285	933	1,201	1,456	1,525	1,622
Cancer	(24)	75th round	_		3,774	4,442	4,416	4,826	7,571	2,312	11,659	5,383	9,777	10,395
Cancer	(23)	75th round	_		36,673		43,156	76,789		50,830		79,562	1,35,906	
NCD	(34)	71st round	Rural	Private	19,245		22,860	29,610	43,129	591		519	676	833
				Public	7,130		6,612	11,366	15,223	294		379	538	549
			Urban	Private	29,607		28,923	43,826	69,239	565		934	807	1,080
				Public	6,047		9,235	11,696	21,479	186		417	465	524
Cancer	(40)	75th round	_		54,763	96,798	79,751	77,802	1,18,700	_	_	_	_	_
Cancer	(19)	71st round	Private		_	48,083	48,857	92,169	95,422	_	_	_	_	_
			Public		_	27,308	24,226	27,138	34,638	_	_	_	_	_
Diarrhea	(21)	71st round	_		5,805		4,445	6,648		_	_	_	_	_
Fever					6,815		8,173	10,246		_	_	_	_	_
Cataract					4,208		5,823	18,514		_	_	_	_	_
Tuberculosis					12,304		9,407	21,387		_	_	_	_	_
Respiratory diseases					9,996		11,721	19,941		_	_	_	_	_
Asthma					8,650		9,060	23,396		_	_	_	_	_
Hypertension					9,665		12,255	20,079		_	_	_	_	_
Diabetes					9,413		13,430	18,756		_	_	_	_	_
Jaundice					12,145		19,301	22,920		_	_	_	_	_
Gastro-intestinal diseases					13,238		15,972	27,156		_	_	_	_	_
Neurological diseases					14,236		15,722	27,843		_	_	_	_	_
Musculoskeletal diseases					15,820		20,399	30,454		_	_	_	_	_
Genitourinary diseases					16,031		19,067	34,771		_	_	_	_	_
Injuries					18,464		20,408	38,959		_	_	_	_	_
Heart diseases					21,180		25,263	63,729		_	_	_	_	_
Cancer					45,538		50,033	70,190		_	_	_	_	_
All diseases					12,391		15,777	30,370		_	_	_	_	_
Communicable diseases					7,784		9,598	16,180		_	_	_	_	_
NCDs					17,690		21,995	41,976		_	_	_	_	_
Inpatient survivors	(14)	52nd round	_		2,420		3,744	13,102		_	_	_	_	_
Inpatient decedents					1,654		5,346	22,415		_	_	_	_	_
Inpatient survivors		60th round			9,921		13,242	23,793		_	_	_	_	_
Inpatient decedents					14,161		20,181	37,247		_	_	_	_	_
Inpatient survivors		71st round			12,063		15,165	28,884		_	_	_	_	_
Inpatient decedents					29,286		28,032	70,886		_	_	_	_	_
Inpatient survivors	(18)	60th round	_		4,563		6,150	10,946		_	_	_	_	_
Inpatient decedents					6,447		8,801	16,424		_	_	_	_	_

*Median OOPE.

Poorer households’ resort to distress financing methods like borrowing compared to wealthier households, who rely more heavily on savings or income for healthcare expenses (8). Furthermore, a distinct rural–urban divide exists, with the incidence of distress financing being considerably higher among the rural poor compared to their urban counterparts (14, 15, 18, 19, 21, 23–25, 28–31, 34–36, 39, 40).

### 3.5 Components of OOPE

Twelve studies within this review disaggregated OOPE into their constituent components as shown in Table 7. The constituent components of OOPE were disaggregated, resulting in the categorization of OOPE as direct medical expenses (e.g., doctor’s fees, medication costs, diagnostic tests, bed charges) and indirect medical expenses (e.g., transportation costs associated with hospitalization or outpatient visits). OOPE components differed by healthcare provider type, age groups, the potential influence of place of residence. There is a significant increase in the proportion of the Indian population reporting any form of OOPE from approximately 60% during 1993–1994 to 80% in 2011–2012. The increase in OOPE, specifically for medicines, surpassed 70% during this timeframe. Data from 2011 to 2012 indicates that over 11 million (4%) Indian households incurred OOPE exceeding 25% of their total household expenditure. Among these, more than 4.4 million households incurred such payments solely for medication purchases. A lower threshold of 10% of total household expenditure reveals a more concerning scenario. An estimated 46 million households faced financial hardship due to healthcare costs, with 29 million households experiencing such hardship solely due to OOP payments for medicines. When considering non-food expenditure as a measure of basic living standards, a similar pattern emerges. In 2011–2012, a significant proportion of households incurred OOP payments for medicines, with such payments reaching as high as 40% of their non-food expenditure. The analysis reveals that average monthly OOP payments for medicines were consistently higher for outpatient care compared to inpatient care across key disease conditions. This disparity, coupled with a potentially higher frequency of outpatient visits compared to inpatient stays, may contribute to a higher incidence of financial hardship (41). Despite the mandate of free maternal services in public healthcare, OOPE for maternal care remains a significant burden too, primarily incurred for medications and diagnostic procedures (42). In public health centers, the largest proportion of OOPE (36%) was allocated to unspecified “other” categories, followed by medicine (26%), transportation, and hospital stay (13% each), and tests (11%). Conversely, private healthcare centers allocated the highest proportion of OOPE to hospital stays (34%), followed by medicine (19%), tests (16%), others (22%), and transportation (9%) (14, 18–20, 22, 23, 28–30, 34, 35, 39, 40, 43, 44).

**Table 7:** Components of OOPE.

Disease condition	Study ID	NSSO round	Sector	Average expenditure per hospitalization (In ₹)							Average expenditure per out-patient visit (In ₹)					
				Direct medical expenditure					Indirect expenses		Direct medical expenditure				Indirect expenses	
				Doctor/surgeon fee	Medicines	Diagnostics	Bed charges	Others	Transport	Others	Doctor/surgeon Fee	Medicines	Diagnostics	Others	Transport	Others
Child delivery care	(35)	71st round	Private	16,937					620	1,173	_				_	_
			Public	1,697					401	669	_				_	_
Child delivery care	(39)	71st round	Private	21,675*					609*	1,024*	_				_	_
			Public	2,540*					450*	660*	_				_	_
Hypertension	(28)	75th round	Private	25,326					727	1,493	534				29	15.5
				3,515	5,182	2,595	2,871	1,669			79	199.5	39	16		
			Public	2,473					402	696	219				37	24
				92	1,455	488	55	213			11	95	13	6		
			NGO	19,725					402	1,777	428				45	13
				3,478	4,161	1,176	2,051	2,188			42	115.75	155	0.2		
NCD	(30)	75th round	Private	47,457*					1,239*	2,547*	_	_	_	_	_	_
			Public	10,549*					875*	1,747*	_	_	_	_	_	_
Non - NCD			Private	29,579*					865*	2,017*	_	_	_	_	_	_
			Public	4,632*					534*	1,079*	_	_	_	_	_	_
Rheumatic diseases	(20)	71st round	Private	5,713	6,226	2,487	3,158	2,014	819	1,820	101	556	84	40	70	43
			Public	715	3,186	1,114	334	661	481	1,257	6	203	27	10	40	24
Mental health disorders	(29)	75th round	Private	4,423	11,987	3,687	4,923	2,273	1,100	1,935	169	1,091	380	790	183	138
			Public	54	3,958	1,199	164	557	1,200	1,662	2	438	19	22	62	47
Cancer	(23)	75th round	_	51,657					5,230		81,595				12,204	
NCD	(34)	71st round	Rural	Private	_	7,021*	_			_		_	294*	_		_	
				Public	_	3,508*	_			_		_	453*	_		_	
			Urban	Private	_	8,100*	_			_		_	588*	_		_	
				Public	_	3,789*	_			_		_	270*	_		_	
Cancer	(44)	75th round	Q1#	6,411	15,980	5,133	2,781	5,738	1,634	4,407	_	_	_	_	_	_
			Q2#	14,226	35,152	13,311	11,904	9,422	2,084	6,559	_	_	_	_	_	_
			Q3#	9,954	21,374	7,363	5,436	9,401	2,812	5,960	_	_	_	_	_	_
			Q4#	6,735	26,318	11,991	7,934	4,996	2,528	6,100	_	_	_	_	_	_
			Q5#	12,162	32,831	10,359	6,672	6,843	3,539	5,938	_	_	_	_	_	_
Cancer	(19)	71st round	Private	29,066					_	_	_	_	_	_	_	_
			Public	24,523					_	_	_	_	_	_	_	_
Cancer	(22)	75th round	_	_	18,670*	6,659*	_	_	5,714*		_	2,216*	372*	_	396*	
High – expenditure chronic ailments			_	_	6,079*	2,874*	_	_	2,771*		_	419*	57*	_	66*	
Other chronic ailments			_	_	3,386*	1,449*	_	_	1,839*		_	407*	78*	_	87*	
All chronic ailments			_	_	3,857*	1,649*	_	_	1,978*		_	412*	74*	_	81*	
Delivery care	(43)	71st round	_	1,669	1,733	662	720	533	494	878	_	_	_	_	_	_
		75th round	_	1,624	1,770	769	712	601	512	926	_	_	_	_	_	_
Inpatient survivors	(14)	60th round	_	15,485							_	_	_	_	_	_
				4,262	4,644	1,552	1,709	3,314	656	1,366						
			0–15**	8,306							_	_	_	_	_	_
				1,779	2,776	884	1,260	2,041	415	896						
			15–59**	16,848							_	_	_	_	_	_
				4,946	5,154	1,680	1,821	3,577	727	1,469						
			≥60**	18,374							_	_	_	_	_	_
				4,649	5,010	1,696	1,793	3,682	660	1,488						
Inpatient decedents			_	22,649							_	_	_	_	_	_
				7,673	9,404	1,992	2,103	4,295	1,525	2,795						
			0–15**	12,775							_	_	_	_	_	_
				3,008	3,769	1,472	1,676	3,329	543	1,084						
			15–59**	25,288							_	_	_	_	_	_
				7,816	9,642	2,046	2,162	4,729	1,201	2,608						
			≥60**	24,252							_	_	_	_	_	_
				8,731	10,509	2,008	2,273	4,172	2,427	3,761						
Inpatient survivors		71st round	_	19,438							_	_	_	_	_	_
				5,193	5,307	2,471	3,087	2,532	675	1,483						
			0–15**	11,911							_	_	_	_	_	_
				3,089	3,319	1,524	2,381	1,277	484	1,246						
			15–59**	19,590							_	_	_	_	_	_
				5,234	2,463	2,475	2,957	2,324	703	1,498						
			≥60**	24,450							_	_	_	_	_	_
				6,775	6,473	3,188	4,010	3,991	737	1,614						
Inpatient decedents			_	43,897							_	_	_	_	_	_
				12,962	14,543	6,842	8,892	5,515	1,440	2,492						
			0–15**	32,897							_	_	_	_	_	_
				7,241	13,638	8,283	5,146	7,072	1,349	2,528						
			15–59**	53,599							_	_	_	_	_	_
				14,108	20,259	8,158	11,349	6,965	1,832	3,216						
			≥60**	38,751							_	_	_	_	_	_
				13,023	11,010	5,810	7,965	4,367	1,169	1,992						
Inpatient survivors	(18)	60th round	All	6,885					563		_	_	_	_	_	_
				2,094	2,266	_	_	1,629	327	417						
			Private	8,916					615		_	_	_	_	_	_
				2,249	2,606	_	_	1,948	360	463						
			Public	3,651					484		_	_	_	_	_	_
				1,123	1,835	_	_	974	276	350						
Inpatient decedents			All	10,134					932		_	_	_	_	_	_
				3,610	4,407	_	_	2,013	701	405						
			Private	13,550					1,266		_	_	_	_	_	_
				4,416	6,872	_	_	3,098	1,005	439						
			Public	6,571					632		_	_	_	_	_	_
				714	2,681	_	_	762	436	371						

*Values explicitly stated as OOPE, #MPCE Quintiles, **Age groups.

### 3.6 Age

Nine studies explored the relation between OOPE and age of patients as in Table 8. The relationship between OOPE and the age of patients was explored, a general upward trend in OOPE with increasing age was observed, and the association exhibited heterogeneity across studies.

**Table 8:** OOPE by disease/condition and age.

Disease condition	Study ID	Survey	Sector	Average OOPE per hospitalization (In ₹)					Average OOPE per out-patient visit (In ₹)				
				Age group (in years)					Age group (in years)				
				0–14	15–35	36–59	≥60						
NCD	(15)	60th round	_	18,723	26,246	28,264	27,171		_	_	_	_	
		71st round		24,705	25,173	29,603	33,342		_	_	_	_	
		75th round		22,965	24,438	30,435	35,394		_	_	_	_	
				15–24	25–29	30–34	>34						
Child delivery care	(39)	71st round	Private	15,940	16,961	19,976	53,349		_	_	_	_	
			Public	3,359	3,675	4,079	4,035		_	_	_	_	
				15–20	21–25	26–30	≥31						
Institutional delivery	(36)	NFHS - 5	Private	25,339*	24,659*	25,833*	27,026*		_	_	_	_	
			Public	2,067*	2,095*	2,021*	2,019*		_	_	_	_	
				0–14	15–29	30–44	45–59	≥60	0–14	15–29	30–44	45–59	≥60
Mental health disorders	(29)	75th round	Private	29,035	32,550	35,166	37,330	50,323	2,463	1,051	1,244	1,220	854
			Public	6,975	8,603	10,712	4,881	6,027	544	306	378	650	844
CVD	(25)	75th round	_	4,305	2,372	2,838	3,744		9,438	1,975	1,326	1,351	
Cancer	(24)	75th round	_	5,617	5,291	6,311	7,219		6,902	7,682	7,978	10,156	
Cancer	(23)	75th round	_	47,249	51,068		53,071		64,277	70,702		1,23,042	
				0–5	6–14	15–24	25–59	≥60					
Cancer	(19)	71st round	Private	61,0196	67,044	1,00,445	91,156	71,936	_	_	_	_	
			Public	30,041	36,577	20,947	36,665	19,912	_	_	_	_	
				0–14	15–59		≥60						
Diarrhea	(21)	71st round	_	5,113	5,922		5,193		_	_	_	_	
Fever				7,979	8,735		6,918		_	_	_	_	
Cataract				64,598	7,614		8,851		_	_	_	_	
Tuberculosis				12,904	13,815		10,857		_	_	_	_	
Respiratory diseases				11,003	14,788		15,353		_	_	_	_	
Asthma				8,429	11,666		16,720		_	_	_	_	
Hypertension				15,165	14,311		14,298		_	_	_	_	
Diabetes				10,641	14,480		16,300		_	_	_	_	
Jaundice				11,188	21,236		21,562		_	_	_	_	
Gastro-intestinal diseases				12,872	18,548		20,572		_	_	_	_	
Neurological diseases				14,402	19,206		20,855		_	_	_	_	
Musculoskeletal diseases				25,043	21,777		24,352		_	_	_	_	
Genitourinary diseases				15,863	22,429		32,546		_	_	_	_	
Injuries				16,202	25,085		32,461		_	_	_	_	
Heart diseases				34,241	29,380		52,876		_	_	_	_	
Cancer				47,901	65,070		45,624		_	_	_	_	
All diseases				12,302	18,915		24,640		_	_	_	_	
Communicable diseases				9,077	11,086		11,718		_	_	_	_	
NCDs				21,599	25,523		34,912		_	_	_	_	
Inpatient survivors	(14)	52nd round	_	3,534	8,604		7,600		_	_	_	_	
Inpatient decedents				7,951	16,173		12,431		_	_	_	_	
Inpatient survivors		60th round		8,306	16,848		18,374		_	_	_	_	
Inpatient decedents				12,775	25,288		24,252		_	_	_	_	
Inpatient survivors		71st round		11,897	19,594		24,469		_	_	_	_	
Inpatient decedents				32,897	53,599		38,751		_	_	_	_	
Inpatient survivors	(18)	60th round	_	3,854	7,727		8,514						
Inpatient decedents				5,729	11,257		10,827						
				15–24	25–29		30–49						
Child delivery care	(35)	71st round	_	7,751	8,764		8,969						

*Median OOPE.

Rising healthcare costs for the older adult population pose a significant challenge due to the projected increase in this demographic and the growing burden of chronic illnesses. This concern is amplified by the observation that OOPE per visit often approaches the total cost of treatment. This suggests potential limitations in health insurance as a financial buffer for healthcare needs. The substantial OOP burden can lead to catastrophic healthcare spending and exacerbate poverty, potentially trapping households in a financially precarious situation (16).

The treatment expenditures are demonstrably higher for individuals above 60 years old compared to younger age groups, regardless of income level. This trend can likely be attributed to the presence of multiple chronic conditions (comorbidities) among the older adult, leading to more frequent hospitalizations and longer stays. Additionally, older women tend to spend more on antenatal and postnatal care, while the overall cost of maternity care follows a non-linear pattern, increasing and then decreasing with age (14, 15, 18, 19, 21, 23–25, 29, 35, 36, 39, 40, 45).

### 3.7 Gender

Eight studies examined the association between gender OOPE, as presented in Table 9. The association between gender and OOPE suggested a potential gender disparity in OOPE, with males generally incurring higher costs compared to females. The deviated trend in terms of gender and OOPE suggests the need for further investigation into the factors influencing gender-based differences in healthcare spending.

**Table 9:** OOPE and gender.

Disease condition	Study ID	NSSO round	Sector	Average OOPE per hospitalization (In ₹)		Average OOPE per out-patient visit (In ₹)	
				Male	Female	Male	Female
NCD	(15)	60th round	_	26,778	25,335	_	_
		71st round		37,303	27,144	_	_
		75th round		33,665	24,304	_	_
Mental Health Disorders	(29)	75th round	Private	34,298	41,539	3,047	1,111
			Public	8,964	6,067	636	366
CVD	(25)	75th round	_	3,914	2,407	1,425	1,357
Cancer	(24)	75th round	_	6,069	5,030	9,293	7,947
Cancer	(23)	75th round	_	56,644	46,825	1,03,416	79,479
Cancer	(19)	71st round	Private	1,08,062	70,235	_	_
			Public	27,427	30,835	_	_
Diarrhea	(21)	71st round	_	5,840	5,000	_	_
Fever				8,708	7,367	_	_
Cataract				7,074	11,670	_	_
Tuberculosis				13,615	11,259	_	_
Respiratory Diseases				13,249	13,150	_	_
Asthma				15,415	11,553	_	_
Hypertension				21,242	7,832	_	_
Diabetes				16,796	12,532	_	_
Jaundice				20,025	13,395	_	_
Gastro-intestinal diseases				17,006	18,016	_	_
Neurological diseases				21,941	13,676	_	_
Musculoskeletal diseases				24,015	21,554	_	_
Genitourinary diseases				25,993	20,937	_	_
Injuries				26,227	21,090	_	_
Heart diseases				45,002	27,797	_	_
Cancer				61,935	52,029	_	_
All diseases				20,372	15,477	_	_
Communicable diseases				11,207	9,724	_	_
NCDs				31,233	21,613	_	_
Inpatient survivors	(18)	60th round	_	7,495	6,717	_	_
Inpatient decedents				9,420	11,139	_	_

Disaggregation of OOPE for hospitalization reveals a gender disparity, with males incurring higher costs compared to females. A potential explanation for this discrepancy lies in the prevalence of distress financing (selling assets, borrowing money, or relying on contributions from relatives) for inpatient care in India. Approximately 60% of households resort to such measures, suggesting that financial decisions may prioritize the health of the primary breadwinner over female caregivers, as only 27% of Indian women participate in the formal workforce. This underrepresentation in paid employment, coupled with their role in caregiving, leads to an underestimation of the true cost of healthcare for women (15, 18, 19, 21, 23, 25, 26, 29, 40).

### 3.8 Strategies for coping with OOPE

Four studies investigated the financing mechanisms for OOPE as detailed in Table 10. Investigating the financing mechanisms for OOPE revealed that savings and income were most patients’ primary sources of OOPE financing. Financing healthcare in India displays a significant socioeconomic disparity. A large portion of the population, particularly those in rural areas and lower income quintiles, depend on savings and income to meet OOPE. Lower-income households and those residing in rural areas heavily rely on distress financing mechanisms like borrowing and selling assets. This reliance persists despite a slight decrease over time and disproportionately affects poorer households, trapping them in a cycle of poverty. The data also suggests a rural–urban divide, with rural populations resorting to borrowing at a much higher rate compared to urban areas. This reliance on distress financing is particularly evident for inpatient care compared to outpatient care, highlighting the greater financial burden associated with hospitalization while outpatient care is funded primarily through household savings and income (17, 24, 25, 31, 32, 35).

**Table 10:** Strategies for coping with OOPE.

Disease condition	Study ID	NSSO round	Source of funds for OOPE for hospitalizations (% share)				Source of funds for OOPE for outpatient visits (% share)			
			Savings/income	Borrowing	Sale of Assets	Other	Savings/income	Borrowing	Sale of assets	Other
NCD	(32)	52nd round	46*	30*	_	24*	_	_	_	_
		60th round	45*	34*	_	21*	_	_	_	_
CVD	(17)	61st round	57	35	8	_	_	_	_	_
Diabetes							_	_	_	_
CVD	(25)	75th round	96.7	0.3	0.1	2.9	94.5	1.4	_	4.1
Cancer	(24)	75th round	71.7	16.9	2.7	8.7	92	4.9	0.2	2.9
Child delivery care	(31)	NFHS - 4	81.6	22	3.1	2.9	_	_	_	_
Child delivery care	(35)	71st round	85	11.9	_	3	_	_	_	_

### 3.9 Educational attainment

One study identified a positive correlation between educational attainment and OOPE as given in Table 11. Individuals with higher education levels may incur greater healthcare costs, suggesting a positive correlation between educational attainment and OOPE (21). For many diseases, the costs tend to increase with higher education levels. For instance, the costs for heart disease rise significantly from no education (Rs. 21,922) to higher secondary education (Rs. 66,323). Some diseases show a more pronounced increase in costs with education than others. For example, cancer treatment costs escalate dramatically from Rs. 44,154 for no education to Rs. 93,083 for higher secondary education. In contrast, the increase for respiratory diseases is less steep, moving from Rs. 11,014 to Rs. 22,190 across education levels (21). NCDs generally have higher associated costs compared to communicable diseases. For instance, the costs for diabetes range from Rs. 11,952 for no education to Rs. 18,603 for higher secondary education, while diarrhea costs are lower across all education levels, peaking at Rs. 13,236 for higher secondary education. Individuals with higher education levels may incur higher out-of-pocket expenses, possibly due to access to more advanced treatments or a greater likelihood of seeking care for chronic conditions. Education levels significantly influence OOPE by affecting health literacy, access to care, management of chronic diseases, economic capacity, and the prioritization of preventive health measures. Individuals with higher education levels are often more informed about health issues, treatment options, and preventive measures. This knowledge can lead to better health-seeking behaviors, potentially resulting in higher expenses due to more frequent consultations and advanced treatments. Higher education may correlate with better access to healthcare resources, including private healthcare facilities, which can be more expensive. For instance, the data shows that costs for treatments like cancer are significantly higher in private settings compared to public ones. Educated individuals may be more likely to manage chronic diseases effectively, leading to higher expenditures on medications and regular check-ups. For example, the costs associated with diabetes and hypertension increase with education level, reflecting the ongoing management required for these conditions. This correlation underscores the importance of education in shaping health outcomes and financial burdens associated with healthcare (15, 19, 21, 24, 25, 28, 31, 35, 36, 39).

**Table 11:** OOPE and education level.

Disease condition	Study ID	NSSO round	Sector	Education level			
				No education	Primary	Secondary	Higher secondary
Diarrhea	(21)	71st round	_	4,715	4,154	5,212	13,236
Fever				6,925	7,327	8,900	11,619
Cataract				9,639	8,823	9,420	14,988
Tuberculosis				9,940	12,619	18,764	17,364
Respiratory Diseases				11,014	11,321	15,824	22,190
Asthma				9,246	18,127	11,446	29,102
Hypertension				10,395	15,678	19,335	11,007
Diabetes				11,952	16,171	13,505	18,603
Jaundice				12,629	19,823	24,823	16,040
Gastro-intestinal diseases				14,363	14,058	18,509	27,956
Neurological diseases				14,212	14,135	22,736	31,963
Musculoskeletal diseases				14,213	25,283	28,271	35,360
Genitourinary diseases				18,960	20,057	22,023	37,333
Injuries				19,809	22,117	28,620	29,963
Heart diseases				21,922	38,804	46,776	66,323
Cancer				44,154	61,359	32,414	93,083
All diseases				13,502	16,788	20,309	28,449
Communicable diseases				9,471	9,230	11,088	15,183
NCDs				18,259	27,032	27,981	44,013
Hypertension	(28)	75th round	Inpatient	512	304	296	119
			Outpatient	2,083	1,489	1,586	451
Cancer	(19)	71st round	Private	57,130	93,358	41,202	1,33,020
			Public	23,176	24,760	23,413	42,232
Cancer	(24)	75th round	Inpatient	4,364	3,927	6,944	
			Outpatient	7,170	9,848	9,378	
NCD	(15)	60th round	_	18,280	23,133	48,873	
		71st round		20,266	30,504	51,778	
		75th round		21,172	26,674	41,444	
CVD	(25)	75th round	Inpatient	2,167	2,596	4,973	
			Outpatient	1,336	1,172	1,597	
Institutional delivery	(36)	NFHS - 5	Private	17,222*	19,554*	25,306*	26,271*
			Public	1,722*	1,754*	2,377*	2,310*
Child delivery care	(31)	NFHS - 4	_	500*	500*	1000*	5000*
Child delivery care	(35)	71st round	_	4,628	5,449	8,890	18,950
Child delivery care	(39)	71st round	Private	35,034	11,779	17,967	24,511
			Public	3,545	3,909	3,325	6,058

*Median OOPE.

### 3.10 OOPE and institutional deliveries

An investigation into the relationship between institutional delivery and OOPE revealed consistently lower OOPE for deliveries occurring in public hospitals compared to private facilities (Tables 12, 13). This finding held true even when considering Caesarean sections. Food and travel expenses constitute a larger proportion of total delivery costs in public healthcare facilities compared to private facilities. Specifically, these expenses account for 29.6% of normal delivery costs and 21.1% of caesarean delivery costs in public hospitals. In contrast, food and travel expenses represent a smaller proportion of total delivery costs in private healthcare settings, at 11.1 and 8.5% for normal and caesarean deliveries, respectively. Individuals relying solely on savings for the procedure incurred the lowest mean OOPE. Conversely, the mean OOPE was highest for those who financed the delivery through a combination of savings, asset sales, and borrowing. This suggests that utilizing multiple financing sources, particularly debt or asset liquidation, significantly increases the total financial burden associated with caesarean deliveries. Private sector hospitalizations were significantly more expensive than the public sector, with a ninefold difference in OOPE. Having a second or subsequent child generally results in lower maternity expenses. From 2004 to 2014, the mean OOPE for comprehensive maternal care (prenatal, natal, and postnatal) increased by 43% in public facilities and 84% in private facilities. Institutional delivery costs rose substantially, with private facilities showing a 76% increase. Educational attainment and economic status were positively correlated with higher OOPE (46). Post-National Rural Health Mission (NRHM), public facility OOPE for comprehensive maternal care increased by 32%, whereas private facility costs were 5.62 times higher than pre-NRHM public facility expenses. These findings underscore the persistent and widening economic burden of maternal healthcare in India, particularly in the private sector, despite the implementation of the NRHM (36, 38, 45, 47–51).

**Table 12:** Mean OOPE for institutional deliveries.

Study ID	Survey	Caesarean		Non-caesarean		All	
		Private	Public	Private	Public	Private	Public
(50)	NFHS - 5	36,594.88	7,304.22	17,633.42	2,582.3	_	_
(51)	NFHS - 5	25,956	5,593	11,241	1,985	18,163	2,541
(36)	NFHS - 5	37,805*	4,429*	17,169*	1,786*	20,132*	2,696*

*Median OOPE.

**Table 13:** Average expenditure on maternal healthcare.

Study ID	Survey	Antenatal		Delivery		Postnatal	
		Private	Public	Private	Public	Private	Public
(48)	60th round	1,162	333	6,720	2,468	611	303

### 3.11 Health insurance

The impact of health insurance on OOPE was explored in four studies, as detailed in Table 14.

**Table 14:** Average OOPE per hospitalization by type of insurance and facility.

Health insurance status	Study ID	Private facility	Public facility	Total
Publicly funded health insurance	(52)	23,361	3,846	12,999
Government employer		28,515	5,085	20,505
Private employer		21,219	3,677	16,724
Private voluntary health insurance		27,702	6,364	25,921
Not insured		29,478	4,122	16,171
Other		18,301	4,214	12,682
Publicly funded health insurance	(51)	17,627	2,235	_
Not insured		18,327	2,635	_
Overall insured	(55)	13,432		
Government sponsored health insurance		11,487		
Uninsured		14,938		
Private voluntary health insurance		24,258		
Government funded insurance scheme	(53)	19,737	3,987	12,408
Others		20,764	7,934	18,510
Uninsured		24,341	5,437	15,647
Government health insurance	(56)	15,464		
General health insurance		16,018		
Private health insurance		25,201		
Uninsured		20,496		

Health insurance appears to increase overall hospitalization rates (6.2 vs. 4.6% for insured vs. uninsured) but demonstrates limited effectiveness in reducing OOPE, particularly when seeking treatment at private facilities that are often preferred despite higher costs. Employer-sponsored insurance is more effective than government-funded schemes in mitigating the OOPE burden. This highlights potential shortcomings of the latter, such as a lack of cashless transactions and limited impact on private healthcare costs. While the stated objective of most government-funded insurance schemes is to facilitate cashless hospitalization rather than merely reducing OOPE, empirical evidence suggests a significant gap in service delivery. Only 2.8% of hospitalizations among insured individuals benefited from cashless services, compared to 1.5% among the uninsured population. Analysis of NFHS 4 and NFHS 5 data reveals a limited correlation between OOPE and health insurance coverage for caesarean sections in public health facilities. Substantial disparities in OOPE and insurance coverage exist within states. This variation may be attributed in part to the suboptimal performance of public financial health insurance (PFHI) schemes, characterized by delays in reimbursements and low claim settlement rates. Furthermore, economic disparities persist despite insurance coverage. Hospitalization rates increase with socioeconomic class, irrespective of insurance status. This suggests deeper issues within the public health system, potentially driving individuals toward private providers, even with high OOPE. The burden remains disproportionately high for low-income households, regardless of insurance status. Overall, the findings suggest that current health insurance models in India may require improvement to ensure broader financial protection and address existing socioeconomic inequalities in healthcare access.

Significant disparities exist in insurance claim reimbursement among Indian women for child delivery care. Factors such as age, education, urban residence, wealth, religion, household size, and location influence claim and reimbursement rates. Older, educated, urban, and wealthier women tend to have higher claim amounts but still face substantial shortfalls. Private healthcare providers offer significantly better reimbursement rates compared to public facilities. Despite claims, only 66% of the total amount was reimbursed, indicating a persistent financial burden on insured women (42, 51–56).

## 4 Discussion

This review examines the multifaceted nature of OOPE in the Indian healthcare system. It delves into the interplay between various socio-demographic factors and economic considerations that influence OOPE. These factors include: the source of healthcare (public vs. private) and the specific disease or condition being treated; place of residence (urban vs. rural); socioeconomic status; the breakdown of OOPE components (medications, diagnostics, etc.); age and gender of the patient; coping mechanisms employed to manage OOPE; educational attainment; the association between OOPE and institutional deliveries; and finally, the role of health insurance in mitigating OOPE. By analysing these interrelated elements, the review aims to provide a comprehensive understanding of the complex landscape of OOPE in India.

OOPE for NCD treatment has increased dramatically since the mid-1990s (32). A total 40–50% of these costs are financed through precarious measures like household borrowing and asset sales, highlighting the substantial financial vulnerability associated with NCDs. Hospitalization for NCDs poses a greater financial risk compared to communicable diseases. Households with NCD-related hospitalizations face a higher likelihood of catastrophic spending and impoverishment. Public hospitals show a trend of NCD-affected households incurring more than double the OOPE compared to non-NCD households (27). A significant portion of NCD-related expenses are attributed to essential elements like medications, diagnostics, and medical appliances. Medicines constitute a substantial portion of healthcare expenditures (both inpatient and outpatient care) across public and private facilities. The inadequate availability of free or subsidized essential drugs in public health facilities forces individuals to purchase medicines from open markets, leading to higher OOPE or forgone treatments. Affordability remains a critical issue, with a significant proportion of Indian households unable to afford necessary medications, particularly in rural areas and among lower-income groups. The situation is exacerbated by factors such as unaffordable prices, reliance on foreign-made drugs, and problematic alliances between doctors and foreign manufacturers. To address these challenges, the Indian government has initiated programs like the Pradhan Mantri Bhartiya Janaushadhi Pariyojana to provide access to affordable generic medicines. However, there is a pressing need to improve drug procurement and supply chain systems in public health facilities and to promote the adoption of generic medicines to reduce the financial burden of NCDs like diabetes on Indian households (26, 34, 57).

While higher-income households allocate a larger share of their expenditure to OOPE, lower-income households are more susceptible to falling below the poverty line due to even minor healthcare expenses. In cases of multimorbidity, the treatment of high-cost conditions such as cancer and cardiovascular diseases is often underfinanced, particularly in outpatient settings (40). This underfinancing may be due to budget constraints, utilization of lower-cost treatment options, or the severity of the condition leading to a shift toward home care. NCD-related hospitalization expenses in India are particularly catastrophic for the poorest quintile, with cancers, psychiatric and neurological disorders, and injuries being the most financially burdensome conditions, especially when care is sought in the private sector.

Private facilities generally entail much higher OOPE than public facilities, often 3–5 times greater than public facilities for both inpatient and outpatient care. This disparity is attributed to several factors, including better infrastructure, quality of services, and the use of advanced medical technology in private hospitals. Despite the higher costs, many individuals, especially those from higher socioeconomic groups, prefer private facilities due to perceived better quality and availability of services (17).

The public sector, while more equitable in terms of utilization among lower socioeconomic groups, faces challenges such as inadequate infrastructure at primary healthcare levels, concentration of specialized facilities in urban areas, and significant OOPE for drugs and diagnostics. These factors often lead to difficulties in accessing appropriate care, particularly for rare diseases and severe illnesses, resulting in individuals resorting to private care despite the financial burden (16, 20).

The private sector plays a crucial role in providing health services, especially for NCDs and cancer treatment. However, the profit-maximization nature of private healthcare and differential charging schemes often lead to catastrophic health expenditures for many households. This situation underscores the need for comprehensive healthcare reforms in India, including better regulation of the private sector, improvement of public healthcare infrastructure, and the development of more inclusive health insurance mechanisms to reduce the financial burden on households, particularly those from lower socioeconomic backgrounds (58).

The burden of OOPE is quite often disproportionately distributed among population subgroups, with households having multiple older adult members experiencing greater financial strain in both public and private settings, likely due to the prevalence of multiple health conditions among the older adult. Urban residents face higher OOPE compared to their rural counterparts, possibly due to elevated treatment costs in urban areas. This suggests that social factors significantly impact healthcare-seeking behavior and spending patterns. Household economic status was observed to have a direct correlation with OOPE in healthcare financing, reflecting the ability-to-pay principle (30).

This research highlights the significant financial burden imposed by CVDs on Indian households, particularly those of lower socioeconomic status. The economic impact of CVDs is multifaceted, manifesting in higher OOPE and reduced non-medical spending. Lower-income households are especially vulnerable, often resorting to borrowing and asset sales to cope with these financial pressures while also experiencing a more pronounced decline in workforce participation. A large proportion of individuals hospitalized for CVDs belong to the economically productive age group, potentially weakening household financial stability due to lost earnings. Concerning, the poorest quintile shows the lowest rates of hospitalization and outpatient care for CVDs, likely due to financial constraints rather than lower disease prevalence. This behavior may exacerbate existing health conditions and perpetuate the cycle of poverty. The research also found that the highest OOPE for both hospitalization and OPD care for CVDs occurred in the 0–14 years age group, possibly due to the need for specialized interventions for pediatric structural heart defects. While absolute OOPE is lower for poorer quintiles, the healthcare burden as a proportion of total consumption expenditure is higher for these groups compared to the richest quintile. These findings underscore the need for effective health insurance mechanisms and policies supporting employment opportunities and income security for low-income households to mitigate the economic hardship caused by CVDs in India (59).

Long treatment protocols including radiotherapy, chemotherapy, and sophisticated diagnostics are the primary reasons for expensive cancer care. These expenses are further accentuated by the poor geographical dispersion of cancer treatment facilities, forcing patients to incur travel and boarding expenses to seek care at specialist oncology facilities. The absence of prepayment and risk-pooling mechanisms further increases the financial burden. This research underscores the substantial financial burden associated with cancer treatment in India, attributing it primarily to lengthy treatment protocols, sophisticated diagnostics, and poor geographical distribution of cancer care facilities. Financial hardship is particularly acute among the poorest quintile, rural residents, and less educated individuals. The analysis emphasizes the need for targeted expenditure support schemes for cancer patients and highlights the importance of prevention and early screening initiatives. With 75% of patients diagnosed at advanced stages and 30–50% of cancers potentially preventable, integrating cancer screening protocols into primary health centers as part of the transition to comprehensive primary healthcare in the public sector would facilitate early detection, improvement of treatment outcomes, and ultimately reduce the cost of care for cancer patients in India (22, 23).

From an equity standpoint, this analysis reveals multifaceted disparities in healthcare financing and access in India. Significant vertical inequity with poorer quintiles bearing a disproportionately higher OOPE burden compared to wealthier segments, indicating a regressive financing system that fails to support the underprivileged adequately. Horizontal inequity is evident between public and private healthcare providers, with private facilities incurring substantially higher costs for patients, exacerbated by inadequacies in the public sector (15, 34). Gender disparities are also apparent, with males exhibiting higher OOPE, potentially due to prioritization of male breadwinners’ health needs in distress financing scenarios (40). Age-related inequities are observed, with individuals over 60 experiencing higher costs due to comorbidities and longer hospital stays. Insurance status plays a crucial role, with uninsured populations facing greater financial burdens, though limitations in coverage can still lead to OOPE for insured patients. The concentration of specialized treatments and surgical procedures in prominent hospitals imposes substantial time and financial burdens on patients, especially those from underserved areas. Geographical disparities are significant, with rural residents incurring higher OOPE due to limited access to quality local healthcare, underscoring the complex interplay of socioeconomic, demographic, and structural factors contributing to healthcare inequities in India (26).

## 5 Conclusion

In conclusion, the analysis of out-of-pocket expenditure (OOPE) in India’s healthcare system reveals a complex landscape characterized by significant inequities and challenges. The burden of non-communicable diseases (NCDs), particularly cardiovascular diseases and cancer, imposes substantial financial pressures on households, with the impact being disproportionately severe for those of lower socioeconomic status. The stark disparities between public and private healthcare sectors, both in terms of cost and perceived quality, further exacerbate these inequities. The concentration of specialized services and personnel in urban areas leaves rural populations at a distinct disadvantage, often forcing them to incur additional expenses for travel and accommodation. The high cost of medications, especially for chronic conditions like diabetes, emerges as a critical factor contributing to the overall financial burden. These findings underscore the urgent need for comprehensive healthcare reforms in India, including strengthening the public healthcare system, improving the regulation of the private sector, expanding insurance coverage, and enhancing the availability and affordability of essential medicines. Additionally, there is a pressing need for targeted interventions to address the specific challenges faced by vulnerable populations, including the older adult, rural residents, and lower socioeconomic groups. Ultimately, addressing these multifaceted challenges will require a concerted effort from policymakers, healthcare providers, and stakeholders to create a more equitable, accessible, and affordable healthcare system for all Indians.

### 5.1 Limitations

One of the key limitations of this study is the unavailability of comprehensive OOPE data for OPD services across several diseases. This data gap restricts the ability to make robust and convincing comparisons between public and private healthcare sectors. Additionally, due to the heterogeneous nature of the data, disease-specific analysis could not be consistently conducted across all conditions. The study aimed to provide an overarching view by presenting available OOPE data for both inpatient (IPD) and OPD services, in estimating indirect costs, only transportation expenses have been considered. Other components of indirect costs, such as accommodation, food, and informal payments were not included due to the lack of consistent data. Future research may benefit from author-wise analysis and categorization of diseases into broader groups to enhance clarity and comparability.

## Data Availability

The original contributions presented in the study are included in the article/supplementary material, further inquiries can be directed to the corresponding authors.

## Abbreviations

NSSO: National Sample Survey Office
NFHS: National Family Health Survey
OOPEs: Out-of-pocket expenditures
UHC: Universal Health Coverage
GDP: Gross domestic product
THE: Total Health Expenditure
GHE: Government Health Expenditure
LMICs: Low- and middle-income countries
GGE: General Government Expenditure
FFC: Fairness of Financial Contribution
CTP: Capacity to pay
NHA: National Health Accounts
IHP: Informal Health Providers
ID: Infectious diseases
NCD: Non-communicable diseases
OOP: Out of pocket
TCE: Total consumption expenditure
MPCE: Monthly *Per Capita* Expenditure
NRHM: National Rural Health Mission
PFHI: Public financial health insurance

## References

[1] Health Systems Governance and Financing (HGF). Global expenditure on health: Public spending on the rise? (2021).

[2] National Health Systems Resource Centre, Ministry of Health and Family Welfare, Macro Graphics Pvt. Ltd. Sharma S Jain M Bose M. National Health Accounts Estimates for India (2019-20). New Delhi: Ministry of Health and Family Welfare, Government of India (2023).

[3] Ambade M Sarwal R Mor N Kim R Subramanian SV. Components of out-of-pocket expenditure and their relative contribution to economic burden of diseases in India. JAMA Netw Open. (2022) 5:e2210040. doi: 10.1001/jamanetworkopen.2022.10040, PMID: 35560051 PMC9107026

[4] Press Information Bureau, Government of India. Economic Survey 2022–23. New Delhi: Press Information Bureau, Government of India (2025).

[5] World Bank Open Data. Current health expenditure (% of GDP) - Low & middle income. Washington, DC: World Bank Open Data (2022).

[6] Patel V Parikh R Nandraj S Balasubramaniam P Narayan K Paul VK . Assuring health coverage for all in India. Lancet. (2015) 386:2422–35. doi: 10.1016/S0140-6736(15)00955-1, PMID: 26700532

[7] Government of India, Ministry of Health and Family Welfare, Statistics Division Rawat A Saha A Kumar A. Rural Health Statistics 2020-21. Government of India, Ministry of Health and Family Welfare, Statistics Division. (2021).

[8] Central Bureau of Health Intelligence, Jaina Offset Printers Mandaviya M Pawar BP Bhushan R Goel A. National Health Profile (2022).

[9] Selvaraj S Karan AK. Deepening health insecurity in India: evidence from national sample surveys since 1980s. Econ Polit Wkly. Central Bureau of Health Intelligence. (2009) 3:55–60.

[10] Gudwani A Mitra P Puri A Vaidya M. Inspiring possibilities, challenging journey. Chicago, IL: McKinsey & company, Inc India Healthcare (2012).

[11] Murray CJ Knaul F Ke X Musgrove P Kawabata K. Defining and measuring fairness in financial contribution to the health system. Geneva: World Health Organization (2000).

[12] Mondal S. Health policy changes and its effect on equity in healthcare financing in India. J Quant Econ. (2018) 16:709–25. doi: 10.1007/s40953-017-0105-4

[13] Singh K Singh SB Ojha A. Ministry of Statistics and Programme Implementation. Sustainable Development Goals - National Indicator Framework Progress Report Ministry of Statistics and Programme Implementation (MoSPI), Government of India. (2023).

[14] Das SK Ladusingh L. Why is the inpatient cost of dying increasing in India? PLoS One. (2018) 13:e0203454. doi: 10.1371/journal.pone.0203454, PMID: 30199546 PMC6130860

[15] Yadav J Allarakha S Menon GR John D Nair S. Socioeconomic impact of hospitalization expenditure for treatment of noncommunicable diseases in India: a repeated cross-sectional analysis of national sample survey data, 2004 to 2018. Value Health Regional Issues. (2021) 24:199–213. doi: 10.1016/j.vhri.2020.12.010, PMID: 33845450

[16] Barik D Arokiasamy P. Rising health expenditure due to non-communicable diseases in India: an outlook. Front Public Health. (2016) 4:268. doi: 10.3389/fpubh.2016.00268, PMID: 27965952 PMC5126101

[17] Rao KD Bhatnagar A Murphy A. Socio-economic inequalities in the financing of cardiovascular & diabetes inpatient treatment in India. Indian J Med Res. (2011) 133:57–63. PMID: 21321420 PMC3100147

[18] Ladusingh L Pandey A. High inpatient care cost of dying in India. J Public Health. (2013) 21:435–43. doi: 10.1007/s10389-013-0572-9

[19] Rajpal S Kumar A Joe W. Economic burden of cancer in India: evidence from cross-sectional nationally representative household survey, 2014. PLoS One. (2018) 13:e0193320. doi: 10.1371/journal.pone.0193320, PMID: 29481563 PMC5826535

[20] Ranjan A Thiagarajan S Garg S Danda D. Progress towards universal health coverage in the context of rheumatic diseases in India. Int J Rheum Dis. (2019) 22:880–9. doi: 10.1111/1756-185X.13488, PMID: 30950207

[21] Kastor A Mohanty SK. Disease-specific out-of-pocket and catastrophic health expenditure on hospitalization in India: do Indian households face distress health financing? PLoS One. (2018) 13:e0196106. doi: 10.1371/journal.pone.0196106, PMID: 29746481 PMC5945043

[22] Goyanka R. Economic and non-economic burden of cancer: a propensity score matched analysis using household health survey data of India. Cancer Res Statistics Treatment. (2021) 4:29–36. doi: 10.4103/crst.crst_6_21

[23] Pandey M Bramhankar M Anand A. Exploring the financial burden due to additional mobility among cancer patients: a cross-sectional study based on National Sample Survey. J Cancer Policy. (2024) 39:100469. doi: 10.1016/j.jcpo.2024.100469, PMID: 38278353

[24] Goyanka R Yadav J Sharma P. Financial burden and coping strategies for cancer care in India. Clin Epidemiol Global Health. (2023) 20:101259. doi: 10.1016/j.cegh.2023.101259

[25] Allarakha S Yadav J Yadav AK. Financial burden and financing strategies for treating the cardiovascular diseases in India. Soc Sci Hum Open. (2022) 6:100275. doi: 10.1016/j.ssaho.2022.100275

[26] Nanda M Sharma R. Financial burden of seeking diabetes mellitus care in India: evidence from a nationally representative sample survey. Health Care Sci. (2023) 2:291–305. doi: 10.1002/hcs2.65, PMID: 38938589 PMC11168574

[27] Karan A Farooqui HH Hussain S Hussain MA Selvaraj S Mathur MR. Multimorbidity, healthcare use and catastrophic health expenditure by households in India: a cross-section analysis of self-reported morbidity from national sample survey data 2017–18. BMC Health Serv Res. (2022) 22:1151. doi: 10.1186/s12913-022-08509-x, PMID: 36096819 PMC9469515

[28] Rajasulochana SR Parthibane S Saravanan E Kumar M Jeyanthi E Gola A . Out-of-pocket expenditure in hypertension related care in India: estimates from national sample survey 2017-18. Asia Pacific J Health Manage. (2023) 18:59–70. doi: 10.24083/apjhm.v18i2.1763

[29] Ranjan A Crasta JE. Progress towards universal health coverage in the context of mental disorders in India: evidence from national sample survey data. Int J Ment Heal Syst. (2023) 17:27. doi: 10.1186/s13033-023-00595-6, PMID: 37726777 PMC10507945

[30] Behera S Pradhan J. Uneven economic burden of non-communicable diseases among Indian households: a comparative analysis. PLoS One. (2021) 16:e0260628. doi: 10.1371/journal.pone.0260628, PMID: 34890400 PMC8664228

[31] Krishnamoorthy Y Ganesh K Sakthivel M Priyan S Rehman T Surendran G. Costs incurred and determinants of out-of-pocket payments for child delivery care in India: evidence from a nationally representative household survey. Int J Health Plann Manag. (2020) 35:e167–77. doi: 10.1002/hpm.2953, PMID: 31709605

[32] Engelgau MM Karan A Mahal A. The economic impact of non-communicable diseases on households in India. Glob Health. (2012) 8:1–10. doi: 10.1186/1744-8603-8-9, PMID: 22533895 PMC3383461

[33] Sangar S Dutt V Thakur R. Rural–urban differentials in out-of-pocket health expenditure and resultant impoverishment in India: evidence from NSSO 71st round. Asia Pac J Reg Sci. (2019) 3:273–91. doi: 10.1007/s41685-018-0095-z

[34] Bose M Banerjee S. Equity in distribution of public subsidy for noncommunicable diseases among the elderly in India: an application of benefit incidence analysis. BMC Public Health. (2019) 19:1–2. doi: 10.1186/s12889-019-8089-y, PMID: 31878911 PMC6933745

[35] Pradhan J Dwivedi R. Do we provide affordable, accessible and administrable health care? An assessment of SES differential in out of pocket expenditure on delivery care in India. Sex Reprod Healthc. (2017) 11:69–78. doi: 10.1016/j.srhc.2016.10.006, PMID: 28159131

[36] Manna S Singh D Ghosal S Rehman T Kanungo S Pati S. Out-of-pocket expenditure and its correlates for institutional deliveries in private and public healthcare sectors in India: findings from NFHS 5. BMC Public Health. (2023) 23:1474. doi: 10.1186/s12889-023-16352-w, PMID: 37532981 PMC10398927

[37] Yadav J Devi S Singh MN Manchanda N. Health care utilization and expenditure inequities in India: benefit incidence analysis. Clin Epidemiol Global Health. (2022) 15:101053. doi: 10.1016/j.cegh.2022.101053

[38] Tripathy JP Shewade HD Mishra S Kumar AM Harries AD. Cost of hospitalization for childbirth in India: how equitable it is in the post-NRHM era? BMC Res Notes. (2017) 10:1–9. doi: 10.1186/s13104-017-2729-z, PMID: 28810897 PMC5556367

[39] Pradhan J Behera S. Does choice of health care facility matter? Assessing out-of-pocket expenditure and catastrophic spending on emergency obstetric care in India. J Biosoc Sci. (2021) 53:481–96. doi: 10.1017/S0021932020000310, PMID: 32583761

[40] Behera S Pradhan J. Economic burden of cancer treatment in India: an equity perspective. J Soc Econ Dev. (2023) 25:334–49. doi: 10.1007/s40847-023-00247-y

[41] Selvaraj S Farooqui HH Karan A. Quantifying the financial burden of households’ out-of-pocket payments on medicines in India: a repeated cross-sectional analysis of National Sample Survey data, 1994–2014. BMJ Open. (2018) 8:e018020. doi: 10.1136/bmjopen-2017-018020, PMID: 29858403 PMC5988077

[42] Kamath R Brand H Nayak N Lakshmi V Verma R Salins P. District-level patterns of health insurance coverage and out-of-pocket expenditure on caesarean section deliveries in public health facilities in India. Sustain For. (2023) 15:4608. doi: 10.3390/su15054608

[43] Balla S Sk MI Ambade M Hossain B. Distress financing in coping with out-of-pocket expenditure for maternity care in India. BMC Health Serv Res. (2022) 22:288. doi: 10.1186/s12913-022-07656-5, PMID: 35241077 PMC8892690

[44] Mohanty SK Panda BK Khan PK Behera P. Out-of-pocket expenditure and correlates of caesarean births in public and private health centres in India. Soc Sci Med. (2019) 224:45–57. doi: 10.1016/j.socscimed.2019.01.048, PMID: 30738236

[45] Goli S Moradhvaj RA Shruti PJ. High spending on maternity care in India: what are the factors explaining it? PLoS One. (2016) 11:e0156437. doi: 10.1371/journal.pone.0156437, PMID: 27341520 PMC4920397

[46] Mishra S Mohanty SK. Out-of-pocket expenditure and distress financing on institutional delivery in India. Int J Equity Health. (2019) 18:1–5. doi: 10.1186/s12939-019-1001-7, PMID: 31238928 PMC6593606

[47] Mohanty SK Kastor A. Out-of-pocket expenditure and catastrophic health spending on maternal care in public and private health centres in India: a comparative study of pre and post national health mission period. Heal Econ Rev. (2017) 7:1–5. 10.1186/s13561-017-0167-1 PMC5603466 28921477

[48] Leone T James KS Padmadas SS. The burden of maternal health care expenditure in India: multilevel analysis of national data. Matern Child Health J. (2013) 17:1622–30. doi: 10.1007/s10995-012-1174-9, PMID: 23114861

[49] Govil D Mohanty SK Narzary PK. Catastrophic household expenditure on caesarean deliveries in India. J Popul Res. (2020) 37:139–59. doi: 10.1007/s12546-019-09236-7

[50] Singh RR Sharma A Mohanty SK. Out of pocket expenditure and distress financing on cesarean delivery in India: evidence from NFHS-5. BMC Health Serv Res. (2023) 23:966. doi: 10.1186/s12913-023-09980-w, PMID: 37679706 PMC10485997

[51] Garg S Tripathi N Bebarta KK. Does government health insurance protect households from out of pocket expenditure and distress financing for caesarean and non-caesarean institutional deliveries in India? Findings from the national family health survey (2019-21). BMC Res Notes. (2023) 16:85. doi: 10.1186/s13104-023-06335-w, PMID: 37217964 PMC10204289

[52] Sharma A. Social health protection and publicly funded health insurance schemes in India: the right way forward? Indian J Labour Econ. (2023) 66:513–34. doi: 10.1007/s41027-023-00445-6

[53] Mahapatro SR Singh P Singh Y. How effective health insurance schemes are in tackling economic burden of healthcare in India. Clin Epidemiol Global Health. (2018) 6:75–82. doi: 10.1016/j.cegh.2017.04.002

[54] Ranjan A Dixit P Mukhopadhyay I Thiagarajan S. Effectiveness of government strategies for financial protection against costs of hospitalization Care in India. BMC Public Health. (2018) 18:1–2. doi: 10.1186/s12889-018-5431-8, PMID: 29661233 PMC5902925

[55] Aashima SR. Is health insurance really benefitting Indian population? Evidence from a nationally representative sample survey. Int J Health Plann Manag. (2024) 39:293–310. doi: 10.1002/hpm.3716, PMID: 37910629

[56] Pandey M Dilip TR. Health insurance as a tool for selecting private hospitalization and mitigating financial burden in India: an analysis of National Sample Survey Data. Glob Soc Welf. (2023) 18:1–3. doi: 10.1007/s40609-023-00320-8, PMID: 40406400

[57] Tripathy JP Prasad BM. Cost of diabetic care in India: an inequitable picture. Diabetes Metab Syndr Clin Res Rev. (2018) 12:251–5. doi: 10.1016/j.dsx.2017.11.007, PMID: 29175198

[58] Tripathy JP Prasad BM Shewade HD Kumar AM Zachariah R Chadha S . Cost of hospitalisation for non-communicable diseases in India: are we pro-poor? Trop Med Int Health. (2016) 21:1019–28. doi: 10.1111/tmi.12732, PMID: 27253634

[59] Karan A Engelgau M Mahal A. The household-level economic burden of heart disease in India. Trop Med Int Health. (2014) 19:581–91. doi: 10.1111/tmi.12281, PMID: 24612174

[60] Sangar S Dutt V Thakur R. Distress financing of out-of-pocket health expenditure in India. Rev Dev Econ. (2019) 23:314–30. doi: 10.1111/rode.12540

[61] Sangar S Dutt V Thakur R. Economic burden, impoverishment, and coping mechanisms associated with out-of-pocket health expenditure in India: a disaggregated analysis at the state level. Int J Health Plann Manag. (2019) 34:e301–13. doi: 10.1002/hpm.2649, PMID: 30230017

[62] Sangar S Dutt V Thakur R. Coping with out-of-pocket health expenditure in India: evidence from NSS 71st round. Glob Soc Welf. (2020) 7:275–84. doi: 10.1007/s40609-019-00141-8

[63] Prasad BM Tripathy JP Thekkur P Muraleedharan VR. Insights from national survey on household expenditure for primary healthcare services availed through informal healthcare providers. J Family Med Prim Care. (2021) 10:1912–6. doi: 10.4103/jfmpc.jfmpc_2274_20, PMID: 34195124 PMC8208194

